# Application of Machine Learning Solutions to Optimize Parameter Prediction to Enhance Automatic NMR Metabolite Profiling

**DOI:** 10.3390/metabo12040283

**Published:** 2022-03-24

**Authors:** Daniel Cañueto, Reza M. Salek, Mònica Bulló, Xavier Correig, Nicolau Cañellas

**Affiliations:** 1 Department of Electronic Engineering and Automation, University Rovira i Virgili, 43007 Tarragona, Spain; danielcanueto88@gmail.com (D.C.); xavier.correig@urv.cat (X.C.); 2 Bruker BioSpin GmbH, Rudolf-Plank-Str. 23, 76275 Ettlingen, Germany; reza.salek@bruker.com; 3 Department of Biochemistry and Biotechnology, Faculty of Medicine and Health Sciences, University Rovira i Virgili (URV), 43201 Reus, Spain; monica.bullo@urv.cat; 4 Institut d’Investigació Sanitaria Pere Virgili (IISPV), Hospital Universitari Sant Joan de Reus, 43204 Reus, Spain; 5 Consorcio CIBER, M.P. Fisiopatología de la Obesidad y Nutrición (CIBERObn), Instituto de Salud Carlos III (ISCIII), 28029 Madrid, Spain; 6 Spanish Biomedical Research Centre in Diabetes and Associated Metabolic Disorders (CIBERDEM), 28029 Madrid, Spain

**Keywords:** automatic profiling, NMR, machine learning

## Abstract

The quality of automatic metabolite profiling in NMR datasets from complex matrices can be affected by the numerous sources of variability. These sources, as well as the presence of multiple low-intensity signals, cause uncertainty in the metabolite signal parameters. Lineshape fitting approaches often produce suboptimal resolutions to adapt them in a complex spectrum lineshape. As a result, the use of software tools for automatic profiling tends to be restricted to specific biological matrices and/or sample preparation protocols to obtain reliable results. However, the analysis and modelling of the signal parameters collected during initial iteration can be further optimized to reduce uncertainty by generating narrow and accurate predictions of the expected signal parameters. In this study, we show that, thanks to the predictions generated, better profiling quality indicators can be outputted, and the performance of automatic profiling can be maximized. Our proposed workflow can learn and model the sample properties; therefore, restrictions in the biological matrix, or sample preparation protocol, and limitations of lineshape fitting approaches can be overcome.

## 1. Introduction

Metabolomic studies characterize the low-molecular-weight components (<1 kDa), called metabolites, in biofluids or cell/tissue extracts [1,2]. The quantification of the metabolite levels in nuclear magnetic resonance (NMR) spectra is called metabolite profiling [3,4,5]. This process requires the measurement of the area below an NMR peak, or signal, that belongs to a metabolite, either by the integration or deconvolution of the signals. In the case of 1D ^1^H-NMR spectra, the most encountered peak shapes can be fitted with a Gaussian and/or a Lorentzian function; they are modelled by a combination of three signal parameters: intensity, chemical shift, and half bandwidth [3,6,7]. The fitting process outputs the combination of parameter values that fits the spectrum lineshape with the lowest error; then, the signal is quantified, integrating the area below the peak shape described by those values.

Several tools have recently been developed, which can automatically estimate signal parameter values [8,9,10]. These tools are usually based on optimization solvers (e.g., the Levenberg–Marquardt algorithm) which evaluate the search space shaped by the range of possible values for each parameter to find an optimal way to represent the replication of the spectrum lineshape with the lowest fitting error [11,12]. Adjustment of the range of values for each parameter will maximize the possibilities of convergence towards an optimal solution, decreasing the possibility of falling into a local minimum.

**Figure 1:**
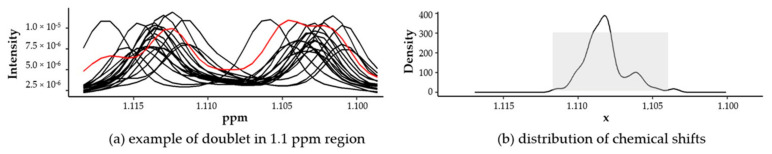
The figure shows a difficult signal fitting where the chemical shift variability present in this signal (**a**) forces lineshape fitting algorithms to consider a wide range (light grey rectangle) of possible chemical shift values during the fitting (**b**).

However, automatic approaches are often compromised by the multiple sources of variability which can be observed in a complex matrix (e.g., macromolecule-based baseline issues, chemical shift, and half-bandwidth variability caused by pH, ionic strength or temperature fluctuations, and signal overlap [13]) (Figure 1a). Therefore, such variability forces the model towards wider ranges of the possible parameter, increasing the chances of falling into a suboptimal solution (Figure 1b) [14]. In addition, the presence of low-intensity signals adjacent to the targeted peak of interest adds complexity to the spectrum lineshape, which can cause suboptimal fittings. Consequently, optimization algorithms may not find the actual parameter values for the signals of interest. As a result, automatic profiling tools sometimes provide wrong metabolite identifications (an important bottleneck in metabolomics [15]) and can perform suboptimal quantifications. To reduce the generation of suboptimal fitting outcomes, several bioinformatic solutions tackle this by reducing the search space during the optimization, for example, the use of a chemical shape indicator [CSI] [16,17], the simultaneous lineshape fitting of all the signals that belong to the same metabolite, or chemical shifts modelling using multiple sources of information, among other methods [18,19]. One should note that such strategies are dependent on prior information. Therefore, they cannot handle unidentified metabolites and might not be robust to small variations in the expected lineshape. For example, simultaneous lineshape fitting is prone to chemical shift variability errors, (please refer to the Supplementary Materials in Section 1 to see additional examples of observed variations, both in the expected half bandwidth and in the expected intensity ratios of metabolite signals in a complex matrix, e.g., see human urine). Consequently, to ensure optimal performance, some tools can only operate optimally in specific matrices or require restrictive procedures in the sample preparation step and/or spectrum acquisition. Such matrix- and protocol-based restrictions can hinder the high-throughput NMR analysis or, worse, can introduce false positives and negatives assignments that can eventually get published in the metabolomics literature when such restrictions are not strictly followed [20,21].

To obtain the best possible quality outcome of lineshape fitting during NMR automatic profiling, one must limit the ranges of possible parameter values, and also adjust the values depending on the biological matrices and sample properties. NMR signals that originated from atoms with similar chemical environments show a similar response to the fluctuations in the sample conditions. As a result, there are extensive multicollinearity in their half-bandwidth and chemical shift values [18]. This property or feature can be exploited to identify signal parameters that do not behave as expected by such multicollinearity. In addition, accurate spectrum-specific predictions with prediction intervals (PIs) for each signal parameter can be estimated according to the information from the collinear signals. Such PIs may be used to create very narrow and accurate value ranges to be used during the lineshape fitting in a subsequent profiling iteration. It is very well known that the intensities of the signals from the same metabolite are correlated. Therefore, the expected signal intensity of different peaks that belong to the same metabolite can be predicted from the estimated known intensities. Consequently, it is not essential to perform the lineshape fitting of all the metabolite signals belonging to the same metabolite simultaneously.

One approach would be to use a prediction protocol that will take advantage of the multicollinearity to limit the ranges of possible parameter values. Machine learning approaches have previously been used in biological applications, mostly for classification. Usually, a training dataset is used to generate the model and an evaluation dataset is used to estimate its performance. Often, different machine learning algorithms are compared in analysis with the aim to select the most suitable method in different applications, such as blood cells [22,23], brain tissue [24,25], or cancer samples [26,27]. In contrast to the existing approaches, we have used machine learning algorithms not to classify or to obtain a result, but to adjust the input parameters (both starting estimates and tolerance) of our lineshape fitting routines. Thus, our proposed prediction workflow is not dependent on prior matrix, protocol, or metabolite information as this information is already encoded in the signal parameter values collected during the first profiling iteration. Therefore, it should be able to better handle an unidentified or atypical metabolite more robustly and is less affected by the properties of the sample matrix- or protocol-based complexities. In addition, we calculate the distance between the predicted parameter values and the ones collected in the initial profiling iteration. The quantified distance may be a better profiling quality indicator than some of those currently in use (e.g., fitting error) and can help to further minimize annotation errors and suboptimal quantifications. To our knowledge, there has been no attempt to provide an open-source flexible automatic signal parameter prediction that maximizes the quality of the information provided by NMR profiling tools. In this study, we show how the proposed workflow helps maximize the quality of metabolite profiling in a 1D ^1^H-NMR dataset.

## 2. Results

### 2.1. H-NMR Metabolite Profiling and Prediction Pipeline of Expected Signal Parameter Values

**Figure 2:**
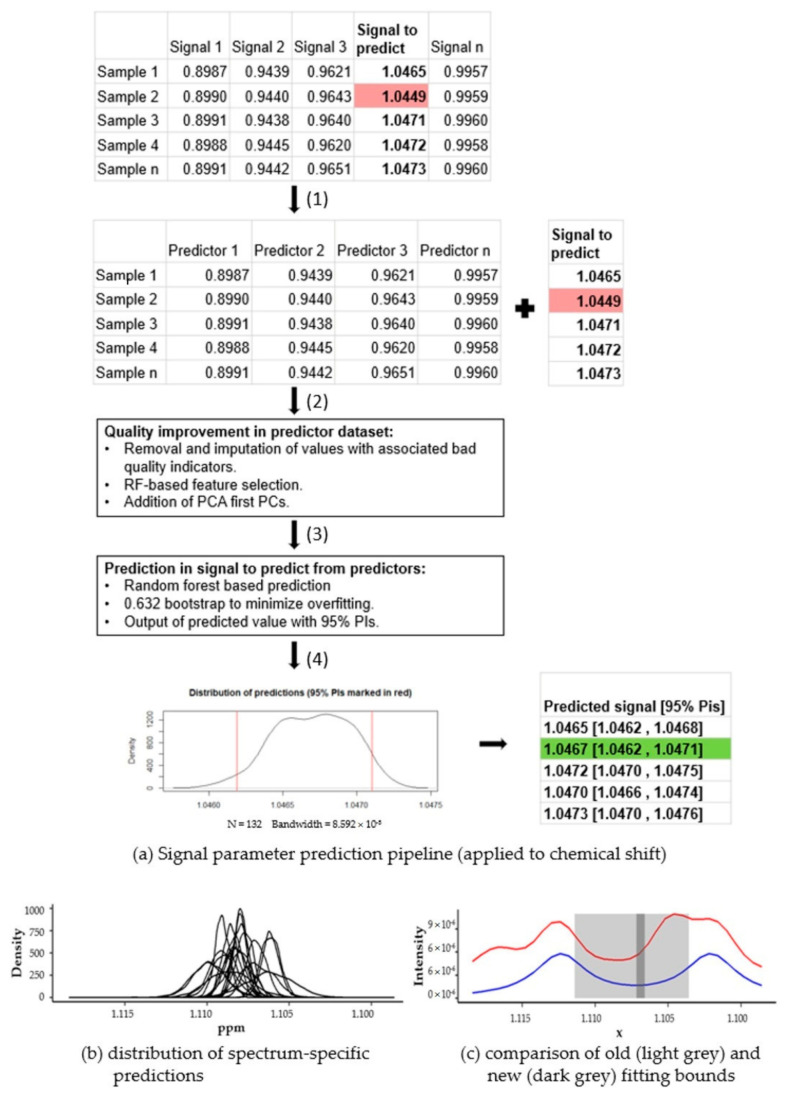
(**a**) In this example, signal parameter prediction pipeline was used to optimize chemical shift value of a signal (table in the upper right). In order to enhance the chemical shifts of the signal in question, a training dataset was built, excluding the signal to predict (**a**,**1**). The dataset is then cleaned, filtered, and enriched to maximize its prediction quality (**a**,**2**). The information from the first iteration was used to train a prediction model. During training, bootstrap resampling was used to avoid overfitting inaccurate values (**a**,**3**). For each predicted chemical shift, the distribution of the predictions made during the bootstrap iterations was built and the median value and 95% PIs of this distribution were outputted (**a**,**4**). After optimization, the predicted value and PIs are shown in the bottom right table; in this case, an inaccurate chemical shift, shaded in red, was clearly outside the 95% PIs, shaded in green. (**b**) shows the distributions of chemical shift predictions generated. These distributions were very narrow and could help generate even narrower chemical shift ranges (dark grey rectangle) than those originally needed without machine learning prediction (light grey rectangle) (**c**).

Automatic metabolic profiling of two 1D ^1^H-NMR datasets was performed using the rDolphin R package [28]. rDolphin is an open-source automatic tool for profiling 1D NMR spectra in a study that collects and exports the signal parameter values for analysis. After NMR spectra profiling, the collected and outputted signal parameters were used to predict, in each signal from different samples, the expected spectrum-specific values (with their PIs) according to the information present and extracted from other spectrum signals (Figure 2a). These predictions can be used in future steps to evaluate the results and to help with identifying inaccurate signal chemical shifts, to improve quantifications or signal and peak fitting.

To make the spectrum-specific prediction of a signal parameter, the values of the parameter extracted from other signals were collected and used to create a dataset of predictors (Figure 2(a1)).

To enhance the quality of the parameter extracted from the dataset of predictors, three common machine learning processes were applied successively (Figure 2(a2)):

- A data cleaning step to minimize the influence of inaccurate feature values (possibly due to wrong annotation or suboptimal quantification) during the prediction phase.
- A feature selection step, using the “Boruta” R package, to filter non-relevant features to reduce the noise in the dataset.
- Finally, we included a further feature engineering step [29], adding the first five PCs of the signal parameter dataset to the predictor dataset. The first PC components explain most of the system variance and relegate noise-related variance to later PCs. Consequently, the possible high noise-related variance in the dataset is minimized, and, hence, prediction performance is enhanced.

After the enrichment steps, a random forest-based prediction model was trained using the enriched dataset of the predictors and used to predict the signal parameter (Figure 2(a3)). The random forest algorithm is an ensemble learning method, based on the bootstrap aggregation (also called bagging) of decision trees [30,31]. The random forest algorithm solves the main drawback of bagging trees (the tendency to create similar decision trees with highly correlated predictions) by adding randomness to the tree construction process. The random forest models show that the possible nonlinear factors have a higher performance match during the exploratory data analysis phase, and a lower variance during the prediction step. In addition, 0.632 bootstrap resampling, a particular bootstrap method that substantially outperforms other alternatives and is a standard option provided by the “caret” R package, was applied to minimize overfitting [32,33] As stated before, we used machine learning algorithms to adjust the input parameters (both starting estimates and tolerance) of rDolphin lineshape fitting routines. As we are interested in the method being applicable to different datasets, without the need to retune the parameters of the machine learning algorithms, we mostly used the default algorithm options without any further optimization.

Then, for each spectrum, the distribution which best represents the predictions generated during the bootstrap (see Figure 2(a4)) was estimated. The spectrum-specific predicted value in the signal parameter analyzed is the median value (with 95% PIs) of this distribution. In the example (Figure 2), one of the signals (shaded in red) was initially not within the calculated prediction interval, but after steps two and three returned to the accepted range (shaded in green).

In case the predictions of parameters were not spectrum-specific, the best possible prediction of the parameter consisted of the median value, found in all spectra having a 95% PIs with 95% central distribution of values, being used. For each signal and parameter, the ranges of the 95% PIs of the spectrum-specific and the spectrum-unspecific predictions were compared to evaluate the results (see Figure 2b).

In addition, a quality indicator, based on the difference between the predicted signal parameters and the parameters obtained during profiling, was calculated. For each one of the signal parameters with available information, the absolute difference was normalized to 0–1. Subsequently, the values obtained for each signal of each spectrum were averaged. As a result, a 0–1 “anomaly score” was generated to show signal parameter anomaly during profiling.

### 2.2. Using Accurate Predicted Values with Narrow PIs That Can Be Used to Maximize Profiling Performance

**Figure 3:**
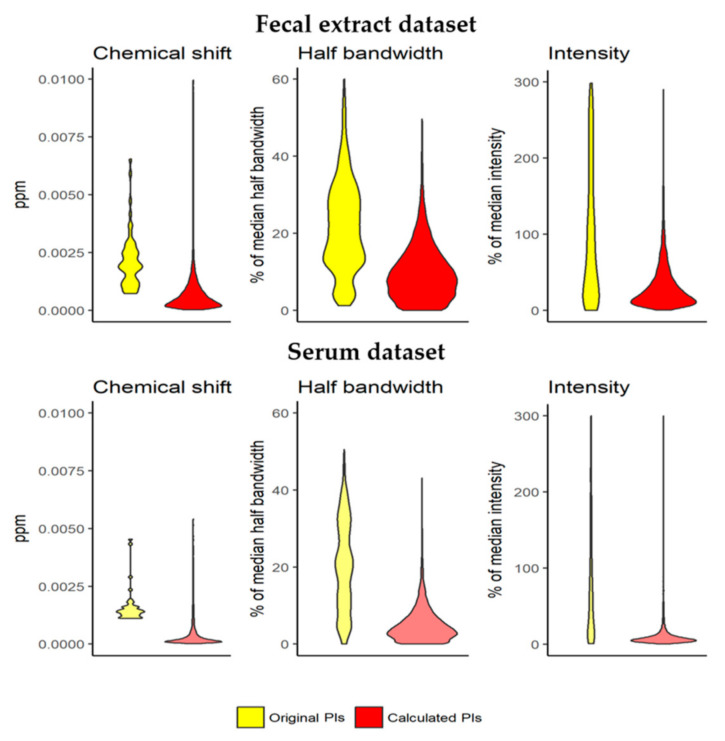
The spectrum-specific 95% PIs of the parameter values PIs are much narrower than the spectrum-unspecific 95% PIs. Chemical shift PIs are generally lower than the bucketing applied (6 × 10^−4^ ppm). The narrow PIs enhance the performance of error minimization algorithms to end in the right local minimum.

The predictions generated (such as the one shown in Figure 2c) have narrow spectrum-specific PIs for all the analyzed signal parameters. For the fecal extract dataset, the median range in the spectrum-specific 95% PIs of the chemical shift was 4.7 × 10^−4^ ppm. This value is lower than the bucket width (6 × 10^−4^ ppm) and is a reduction of 75.8% in the median range in the spectrum-unspecific 95% PIs (1.9 × 10^−3^ ppm) (Figure 3, top left). In the serum dataset, the median range in the spectrum-specific 95% PIs calculated was 1.9 × 10^−4^ ppm, a reduction of 87.1% in the median range in the spectrum-unspecific 95% PIs (1.4 × 10^−3^ ppm) (Figure 3, bottom left).

For half bandwidth, the median range in the spectrum-specific 95% PIs was calculated in the fecal extract dataset at 8.6% of the predicted half bandwidth. This value is a reduction of 58.4% from the median range in the spectrum-unspecific 95% PIs (20.6% of the predicted half bandwidth) (Figure 3, top middle). In the serum dataset, the median range in the spectrum-specific 95% PIs calculated was 4.0% of the predicted half bandwidth, a reduction of 80.3% in the median range from the spectrum-unspecific 95% PIs (20.1% of the predicted half bandwidth) (Figure 3, bottom middle).

For intensity, the median range in the spectrum-specific 95% PIs was calculated in the fecal extract dataset at 22.2% of the predicted intensity. This value is a reduction of 92.8% in the median range in the spectrum-unspecific 95% PIs (309.9% of the predicted intensity) (Figure 3, top right). In the serum dataset, the median range in the spectrum-specific 95% PIs calculated was 6.9% of the predicted intensity, a reduction of 93.3% in the median range in the spectrum-unspecific 95% PIs (102.9% of the predicted intensity) (Figure 3, bottom right). Apart from showing narrow PIs, the predictions also helped to maximize profiling performance when they were used in a new profiling iteration.

The quality of quantifications was ranked using both indicators, the fitting error and the calculated anomaly score. To parameterize its performance (as quality indicators for quantification), this ranking was then used to gradually replace the worst-ranked quantification in each metabolite by the equivalent one obtained in the new higher-quality profiling iteration.

The anomaly score showed an effectiveness at ranking the quantifications which might be further optimized. In comparison with the anomaly score, the fitting error showed a general lower effectiveness to detect improvable quantifications.

## 3. Discussion

The results of the study showed that predicting signal parameter values with the information collected during the initial profiling iteration can help with maximizing the profiling performance. The only limitation is that the number of samples in the set must be high enough (around 30 samples minimum) to ensure the proper functioning of the prediction routines. The improvement achieved in this study has been demonstrated in complex biologically matrices and not in spike-in samples which cannot fully reproduce the usual complexity of metabolomics studies (biofluids). Our study also presents a new quality indicator based on the information generated by our machine-learning-based pipeline. This new quality indicator, called the anomaly score, may provide higher-quality information to improve the detection of suboptimal quantifications and, additionally, enables detection of wrong annotations (a source of false positives and negatives) that can be found in the metabolomics literature [15,20,21]. In addition, our machine-learning-based pipeline (contained in the “signparpred” function in the rDolphin R package) can be exported to other profiling tools.

The great advantage of our approach is in the generation of predictions specific to each signal and each spectrum with accurate and narrow PIs. The high-quality predictions ensure that the algorithmic minimization of the signal fitting error prevents the pervasive problem of falling into wrong local minima when numerous parameter values are optimized (dozens of parameters in the case of complex lineshape fittings). Other approaches try to handle this problem by creating narrow value ranges prior to the profiling. However, when dealing with complex matrices, they may have limitations such as:

- Strict sample preparation requirements or spectrum acquisition limitations. Caveats: difficulty of changing established protocols in laboratories, less flexibility to adapt the spectrum acquisition process to the properties of samples.
- Half bandwidth and chemical shift prediction. Caveats: broadening of TSP signal mediated by protein, nonlinear patterns in certain signals in complex matrices, inability to handle unidentified metabolites [13].
- Simultaneous lineshape fitting of all the signals of a same metabolite. Caveats: variability in the relative intensity of signals depending on the matrix, challenges when the signal chemical shift is not predicted exactly, inability to handle unidentified metabolites.
- Algorithm-based signal alignment. Caveats: signal distortion, wrong annotations [34,35].

In contrast to the above, our approach is not dependent on restrictions or extensive previous information about signal properties; it only needs a flexible first profiling iteration that collects information for accurately characterizing the properties of the metabolite signals profiled. In addition, the information obtained about the signal parameters of unidentified metabolites can be studied to find annotated signals with similar patterns and, consequently, create valuable inferences about their structure and properties.

The maximization of the profiling quality shown in the results was not associated with a correlated decrease in the signal fitting error (the standard quality indicator outputted by NMR profiling tools). The mean fitting error of quantifications increased by 0.26% in the fecal extract dataset and decreased by 0.02% in the serum dataset. This suggests a ceiling in the performance of lineshape fitting approaches when matrices are complex. For example, they may give little importance to the lower intensity signals in the region analyzed, or not fully monitor the high-intensity baseline present, e.g., in the serum. The adjustment information parameterizes the properties of the entire spectral region, considering not only the signals from the metabolites of interest, but also the baseline and the signals from the rest of the metabolites. On the contrary, the information generated by our prediction channel parameterizes the properties of the metabolites, minimizing the influence of the rest of the signals. As a result, the new information generated by this workflow leads to next-generation quality indicators which can, for example, be used to monitor wrong annotations due to the associated chemical shift signal that is not consistent with the information present from the whole dataset. Such quality control means have the potential ability to filter out suboptimal quantifications more effectively. Consequently, it may be possible to profile many more metabolites without decreasing the profiling quality.

The variability of chemical shift is one of the biggest challenges to progress in the automatic profiling of NMR datasets. The PIs achieved during the prediction phase tend to be even lower than the bucket-width chosen. Thanks to the accurate chemical shift prediction, signals can be correctly assigned and the lineshape fitting performance maximized. The fact that chemical shift can be accurately predicted in fecal extract, a matrix with considerable variability in chemical shift and signal overlap, suggests that accurately predicting chemical shift in human urine is achievable. This matrix is of great interest to metabolomics. However, its complexity makes robust automatic profiling a real challenge, and it is recommended that some tools are not used for this type of matrix. A promising technique for maximizing the quality of NMR profiling in human urine through chemical shift prediction has recently been published [18]. Nonetheless, this technique cannot be exported to NMR profiling tools because of licensing restrictions, and it requires strict sample preparation and spectrum acquisition criteria. The machine learning pipeline we propose, when tuned to the special conditions of human urine, may be a generalizable solution to the signal misalignment problem in human urine (please refer to Section 2 of the Supplementary Materials to see the current results for a human urine dataset).

## 4. Materials and Methods

### 4.1. Datasets

For this study, two datasets were analyzed: a dataset of 146 fecal extract samples from a medical treatment study, and a dataset of 212 serum samples from a nutritional intervention study.

For the fecal extract dataset, study participants collected fecal samples at home in sterile fecal collection tubes the same day as, or the day before, their medical appointment and following the standard operating procedures. If required, samples were stored at 4 °C overnight. The fecal samples were stored at −80 °C until processing; details are available in Noguera-Julian, M. et al. [36]. For NMR processing, 70–100 mg of dry fecal matter and 1000 mL of 0.05 M PBS buffer in D2O were placed in a 2 mL Eppendorf tube. The sample was vortexed and sonicated, until complete homogenization, and the mixture was centrifuged (15,000 rpm around 20,000× *g*, 25 min, 4 °C). For NMR measurement, 600 mL of the upper phase were placed into a 5 mm NMR tube and ^1^H NMR spectra were recorded at 300 K on an Avance III 500 spectrometer (Bruker^®^, Ettlingen, Germany) operating at a proton frequency of 500.20 MHz using a 5 mm PBBO gradient probe. One-dimensional ^1^H were acquired using nuclear Overhauser effect spectroscopy. A NOESY pulse program with presaturation was used to suppress the residual water peak, with a mixing time set at 100 ms. The spectral width was 10 kHz (20 ppm), and a total of 256 transients were collected into 64 k data points for each ^1^H spectrum. After zero filling and exponential line broadening (0.5 Hz), spectra were Fourier transformed, manually phased, and baseline corrected using TopSpin software (version 3.2, Bruker BioSpin GmbH, Ettlingen, Germany). Bucketing (6 × 10^−4^ ppm as bucket width) was used, while spectra referenced to TSP at 0 ppm. For the normalization of data, probabilistic quotient normalization [37] was performed through rDolphin [28].

For the serum dataset, sample collection details are available in Hernández-Alonso, P. et al. [38]. For each sample, 300 μL aliquots were mixed with 300 μL of sodium phosphate buffer. The Carr–Purcell–Meiboom–Gill (CPMG) pulse program, with the sample kept at 37 °C, and presaturation was used to suppress the residual water peak. Datasets were acquired on a Bruker 600 MHz Spectrometer (Bruker BioSpin, GmbH, Ettlingen, Germany) equipped with an Avance III console and a TCI CryoProbe Prodigy. The CPMG data were preprocessed on the NMR console (using TopSpin 3.2, Bruker BioSpin, GmbH, Ettlingen, Germany) using zero filling, exponential line broadening (0.5 Hz), and phase correction. Bucketing (6 × 10^−4^ ppm as bucket width) and referencing to the anomer of glucose at 5.233 ppm were performed through rDolphin [28].

### 4.2. ^1^H-NMR Metabolite Profiling Workflow

Automatic metabolic profiling was performed using the rDolphin R package [28]. rDolphin is an open-source tool that collects the values of the signal parameters and exports them for analysis. rDolphin performs a lineshape-fitting-based profiling which adjusts spectral regions to a sum of Lorentzian signals, each one of which is characterized by three parameters: intensity, chemical shift, and half bandwidth. The fitting process is performed using the Levenberg-Marquardt nonlinear least-squares algorithm with lower and upper bounds provided by the “minpack.lm” R package [39]. The values of the algorithm parameters used during lineshape fitting are available in the Supplementary Materials. To avoid falling into local minima, the fitting optimization is iterated a number of times, proportional to the spectrum lineshape complexity. The initial estimated signal parameters are randomly initialized for each iteration. In the next step, the algorithm selects the resolution with the least lineshape fitting error. After lineshape fitting, the areas below the signals are quantified, a specific fitting error for each signal is estimated (procedure explained in Section 4 of the Supplementary Materials), and finally the optimized signal parameter values are collected.

A graphical user interface (GUI) enables the users to select the metabolites target for fitting and the profiling method (area integration, signal deconvolution) for each of the signals is then applied. The GUI can be used to supervise the optimal value ranges for each chemical shift and to change the half bandwidth that can be used during lineshape fitting.

The first dataset 80 signals (66 through deconvolution and 14 through integration) from 52 different metabolites were profiled. For the second dataset, 48 signals (43 fitted through deconvolution and 5 through integration) from 33 different metabolites were profiled. In addition, the signal parameter values and fitting errors were collected for both dataset profiling iterations.

## 5. Conclusions

Most existing NMR profiling tools require some means of restrictions for data analysis parameters to ensure that their workflows can be widely applied to different datasets (e.g., different matrix, different sample preparation method, or the choice for acquisition). In this study, we have demonstrated that, by using previously collected data from the dataset, a more generalized approach to NMR profiling is possible. The nature of multicollinearity that is presented in the collected information enables a narrow prediction of the expected signal parameters, a robust property against the noise present in spectra. As a result, the quality of the signal parameter values derived during the profiling method can be maximized and, therefore, enhance the quality of automatic metabolite profiling.

## Data Availability

rDolphin is an open-source tool available at: http://github.com/danielcanueto/rDolphin (accessed on 6 February 2022).

## Supplementary Materials

The following supporting information can be downloaded at: https://www.mdpi.com/article/10.3390/metabo12040283/s1: Figure S1: variability in the expected intensity, Figure S2: variability in the expected half bandwidths of signals, and Figure S3: spectrum-specific prediction intervals. This is in addition to the values of algorithm parameters used during lineshape fitting, the signal-specific calculations of the lineshape fitting error method, and the analysis of the coefficient of variation after profiling improvement.
