# Supplementary material for: Application of Machine Learning Solutions to Optimize Parameter Prediction to Enhance Automatic NMR Metabolite Profiling
Source: Metabolites. 2022 Mar 24;12(4):283. doi: 10.3390/metabo12040283 (PMC9027668; doi:10.3390/metabo12040283)
Supplement: Supplementary file 1 [file metabolites-12-00283-s001.zip › metabolites-1608646-supplementary.pdf]

Supplementary Materials

Maximizing the Quality of NMR Automatic Metabolite Profiling by a Machine Learning Based Prediction of Fitting Parameters

Daniel Cañueto [1], Reza M. Salek [2], Mònica Bulló [3,4,5,], Xavier Correig [1,4,6] and Nicolau Cañellas [1,4,6*]

[1] Department of Electronic Engineering and Automation, University Rovira i Virgili, 43007 Tarragona, Spain; danielcanueto88@gmail.com (D.C.); xavier.correig@urv.cat (X.C.)
[2] Bruker BioSpin GmbH, Rudolf-Plank-Str. 23, 76275 Ettlingen, Germany; reza.salek@bruker.com
[3] Department of Biochemistry and Biotechnology, Faculty of Medicine and Health Sciences, University Rovira i Virgili (URV), 43201 Reus, Spain; monica.bullo@urv.cat
[4] Institut d'Investigació Sanitaria Pere Virgili (IISPV), Hospital Universitari Sant Joan de Reus, 43204 Reus, Spain
[5] Consorcio CIBER, M.P. Fisiopatología de la Obesidad y Nutrición (CIBERObn), Instituto de Salud Carlos III (ISCIII), 28029 Madrid, Spain
[6] Spanish Biomedical Research Centre in Diabetes and Associated Metabolic Disorders (CIBERDEM), 28029 Madrid, Spain
* Correspondence: nicolau.canyellas@urv.cat

1 .- Variability in the expected half bandwidth and the expected intensity ratios of metabolite signals in complex matrices

Automatic profiling tools must handle the variability in the signal parameters; and typical approaches tend to minimise the search space during the optimization of lineshape fitting. A common method to achieve this minimization is the development of bioinformatics solutions based on empirical observations. The solutions are mostly based on rules that permit the reduction of the search space necessary to consider during the optimization of lineshape fitting.

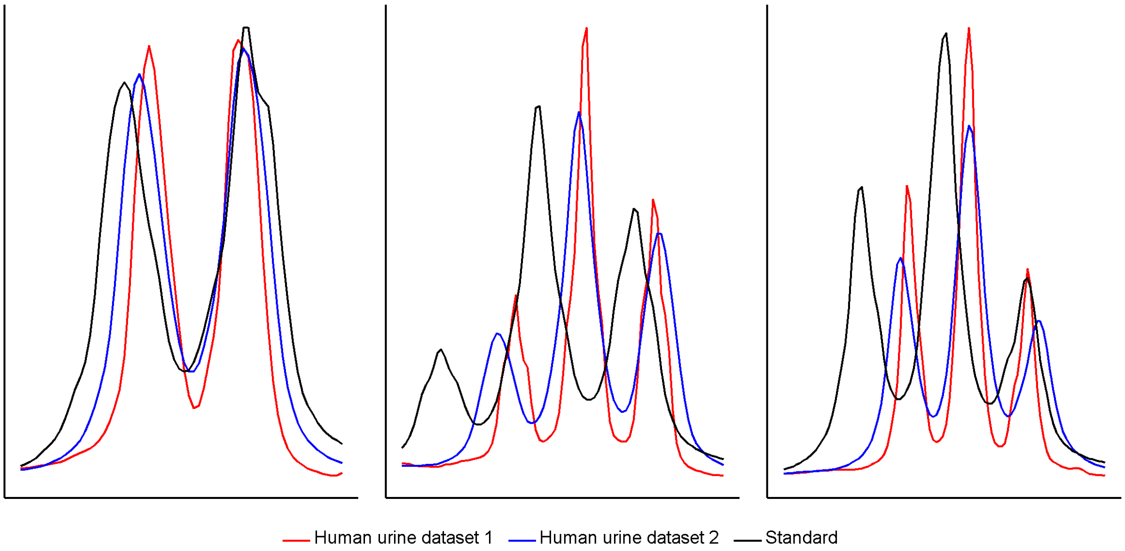

Figure S1. The relative intensities of signals of a same metabolite can be not constant. The three hippurate signals at the 7.85-7.5 ppm region are shown for two datasets of human urine and for the BMRB standard. After normalizing the spectra by the left signal, the other two signals show clear differences in relative intensity even when coming from the same matrix. This variability is mediated by shimming differences and possible other effects related to differences in samples properties or preparation. As a result, the simultaneous lineshape fitting of all metabolites can be compromised as the assumption of constant relative intensity is not accomplished.

Examples of these solutions are the calculation of the half bandwidth of a signal from the half bandwidth of a chemical shape indicator (CSI) or the simultaneous lineshape fitting of all the signals of a same metabolite based on the expected ratios between its signal intensities. However, the assumptions which are based on, can be optimal but are not fully accomplished. For example, internal exploratory analyses of two human urine datasets with different lab protocols show that general assumptions are not present even in the same matrix. More concretely, the relative intensities of the signals of a same metabolite can be different (as shown in the hippurate signals in Figure S1) and the relationships between the half bandwidths of signals and the ones of a CSI such as TSP (as shown in the ratio between a creatinine signal and the TSP one in Figure S2) show differences. Signal lineshapes sometimes do not follow a strict Lorentzian lineshape: Voigt lineshapes (with a % percentage of Gaussian lineshape) might be necessary to fit when shimming variations and other possible kinds of effects appear mediated by sample properties or preparation. The breaking of assumptions can be not observed when doing controlled experiments based on spike-ins but appear when dealing with actual samples from complex matrices. As a result, bioinformatic solutions like the simultaneous lineshape fitting of all the signals of a same metabolite might be not robust to the breaking of these assumptions as the simultaneous lineshape fitting requires a prior strict estimation of chemical shifts, half bandwidths and relative intensities which cannot be ensured.

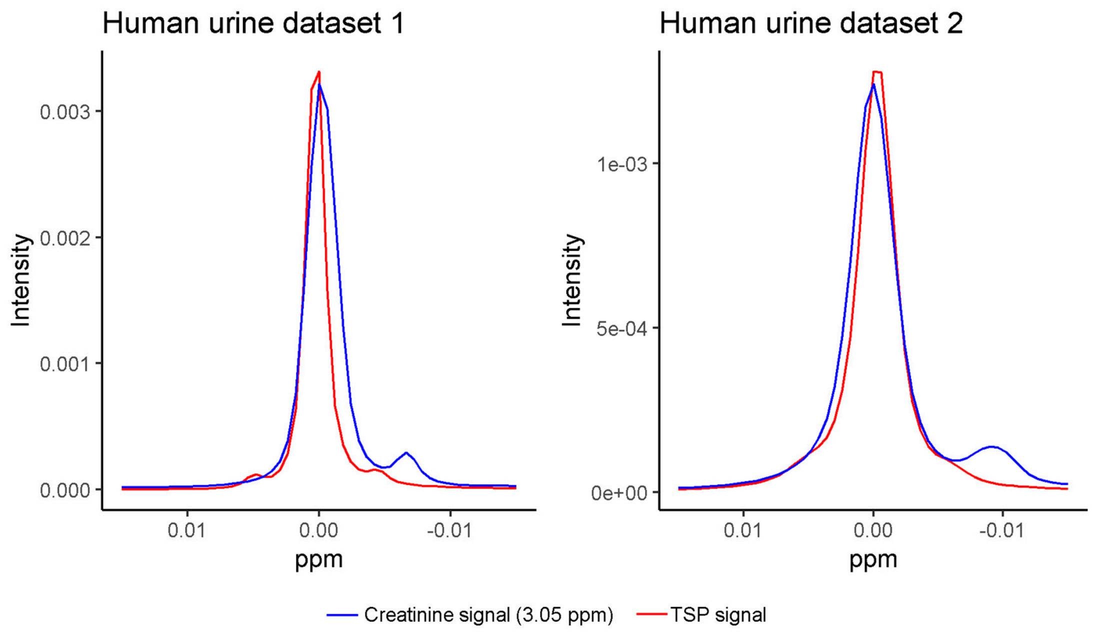

Figure S2. The ratio between half bandwidths of signals can be not constant. The TSP signal is used as CSI to estimate the expected half bandwidth of the rest of signals in a spectrum. However, in datasets of the same matrix (human urine), differences between the ratio of the half bandwidth of a signal such as a creatinine one and the one of the CSI signal can be observed. More concretely, on the dataset 1, the ratio creatinine/TSP is much higher than on the dataset 2. As a result, the assumption of constant ratio between half bandwidths is not accomplished and the estimation of accurate half bandwidths is compromised.

2.- Creation of narrow spectrum-specific PIs in a human urine dataset

For chemical shift, the median range in the spectrum-specific 95% PIs calculated was 5.69e-04 ppm. This value is lower than the bucket width (6e-04 ppm) and is a reduction of 87.16% in the median range in the spectrum-unspecific 95% PIs (4.43e-03 ppm) (Figure S3, left).

For half bandwidth, the median range in the spectrum-specific 95% PIs calculated was 9.66% of the predicted half bandwidth. This value is a reduction of 57.32% in the median range in the spectrum-unspecific 95% PIs (22.62% of the predicted half bandwidth) (Figure S3, middle).

For intensity, the median range in the spectrum-specific 95% PIs calculated was 13.42% of the predicted intensity. This value is a reduction of 92.79% in the median range in the spectrum-unspecific 95% PIs (186.03% of the predicted intensity) (Figure S3, right).

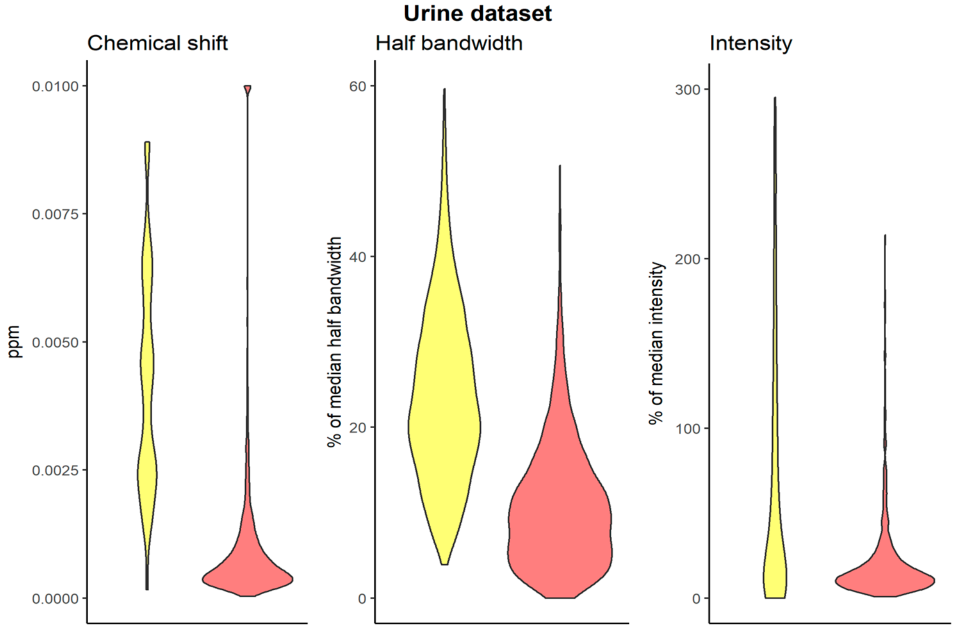

Figure S3. The spectrum-specific 95% PIs of the parameter values. PIs are much narrower than the spectrum-unspecific 95% PIs. Chemical shift PIs are generally lower than the bucketing applied (6e-4 ppm). The narrow PIs enhance the performance of error minimization algorithms to end in the right local minimum.

3.- Values of algorithm parameters used during lineshape fitting

Standard algorithm parameters used during lineshape fitting are available at: https://cran.r-project.org/web/packages/minpack.lm/minpack.lm.pdf. The following parameters were tweaked to maximize quality/speed performance:

- maxiter = 500
- ftol = 1e-6
- ptol = 1e-6
- factor = 0.01

4.- Signal-specific calculation of lineshape fitting error

1. The spectrum region with the 90% central area below the quantified signal is identified.
2. The root mean squared error from the linear model between the spectrum region lineshape and the fitted lineshape is estimated.
3. The root mean squared error is normalized by the maximum of the spectrum region lineshape.

5.- Analysis of coefficient of variation after profiling improvement

The coefficient of variation is a quality indicator of profiling quality (the lower the noise added during profiling, the lower the coefficient of variation). The mean lowering in the coefficient of variation after profiling improvement based on prediction information was 7.8%. In certain metabolite signals, the coefficient of variation decreased more than 25%.
